# Melioidosis in Children, Brazil, 1989–2019 (response)

**DOI:** 10.3201/eid2805.220479

**Published:** 2022-05

**Authors:** Rachel Ximenes Ribeiro Lima, Dionne Bezerra Rolim

**Affiliations:** Municipal Department of Health, Fortaleza, Brazil (R.X.R. Lima);; University of Fortaleza Medical School (R.X.R. Lima, D.B. Rolim); Ceará State University, Fortaleza (D.B. Rolim)

**Keywords:** melioidosis, *Burkholderia pseudomallei*, bacteria, pediatric, melioidosis in children, environmental exposure, melioidosis epidemiology, melioidosis in tropics, Brazil

**In Response**: We would like to respectfully clarify a few points in the comments by Behera et al. (*1*). We did not conclude that the severity of melioidosis in children in Brazil is greater than in other countries; we discussed it as a possibility (*2*). We also discussed that mild to moderate cases are the most prevalent forms in children and that they are underdiagnosed. However, it is possible that the severity of childhood melioidosis in Brazil may be like that in other melioidosis-endemic countries. By emphasizing disease severity, we aimed to draw attention to the detection of melioidosis in children, which can result in high death rates (*3*). Because the severe cases in our study occurred in healthy children, we did not discuss host immunity; this fact does not invalidate the role of immunity in melioidosis pathophysiology. Our objective was the same as that of Wiersinga et al. (*4*).

We described the intense environmental exposure of this age group in our region and recognized the importance of the environment to melioidosis epidemiology. We do not claim that exposure is the only explanation for disease severity, nor that it is a direct cause of severity. Furthermore, we acknowledge that human behavior and habits vary in different regions of the world; for example, tropical areas in which children play outdoors have a higher risk for melioidosis. Currie et al. have recommended additional studies (*5*).

We observed diverse genetic, cultural, and economic factors in the countries where melioidosis is found, whether it is well recognized or not. All of these factors could influence the distribution and severity of the disease (*6*). At this time, we believe a descriptive study can draw attention to melioidosis in tropical regions, such as Brazil and Latin American countries. The goal is to improve detection and reduce deaths from melioidosis in all parts of the world.
